# Relationships between perioperative physical activity and urinary incontinence after radical prostatectomy: an observational study

**DOI:** 10.1186/1471-2490-13-67

**Published:** 2013-12-01

**Authors:** Sean F Mungovan, Bregtje P Huijbers, Andrew D Hirschhorn, Manish I Patel

**Affiliations:** 1 The Clinical Research Institute, Westmead Private Physiotherapy Services, Sydney, Australia; 2 Center for Human Movement Sciences, University Medical Center Groningen, University of Groningen, Groningen, The Netherlands; 3 Urological Cancer Centre and The University of Sydney, Sydney, Australia

**Keywords:** Prostatectomy, Urinary incontinence, Exercise, Pelvic floor muscle training

## Abstract

**Background:**

Higher physical activity levels are continence-protective in non-prostate cancer populations. Primary aims of this study were to investigate changes in physical activity levels over the perioperative period in patients having radical prostatectomy, and relationships between perioperative physical activity levels and post-prostatectomy urinary incontinence.

**Methods:**

A prospective analysis of patients having radical prostatectomy and receiving perioperative physiotherapy including pelvic floor muscle training and physical activity prescription (n = 33). Physical activity levels were measured using the International Physical Activity Questionnaire and/or the SenseWear Pro3 Armband at four timepoints: before preoperative physiotherapy, the week before surgery, and 3 and 6 weeks postoperatively. Urinary incontinence was measured at 3 and 6 weeks postoperatively using a 24-hour pad test and the International Consultation on Incontinence Questionnaire – Urinary Incontinence Short Form (ICIQ).

**Results:**

Physical activity levels changed significantly over the perioperative period (p < 0.001). At 6 weeks postoperatively, physical activity levels did not differ significantly from baseline (p = 0.181), but remained significantly lower than the week before surgery (p = 0.002). There was no significant interaction effect between preoperative physical activity category and time on the 24-hour pad test (p = 0.726) or ICIQ (p = 0.608). Nor were there any significant correlations between physical activity levels and the 24-hour pad test and ICIQ at 3 or 6 weeks postoperatively.

**Conclusions:**

This study provides novel data on perioperative physical activity levels for patients having radical prostatectomy. There was no relationship between perioperative physical activity levels and post-prostatectomy urinary incontinence, although participants had high overall preoperative physical activity levels and low overall urinary incontinence.

## Background

Urinary incontinence is a common complication of radical prostatectomy, with 59-63% of patients experiencing mild to severe incontinence in the early (<6 weeks) postoperative period [1,2]. Pelvic floor muscle (PFM) exercises, commenced preoperatively, have been shown to reduce the severity and duration of PPUI [3-5]. As such, in our clinical setting, patients having radical prostatectomy are routinely referred to physiotherapy for pre- and postoperative PFM training [6]. While multiple other factors influence the severity and duration of post-prostatectomy urinary incontinence (PPUI) [7], most are non-modifiable, e.g. patient age [8], or are related to the surgical technique, e.g. ‘nerve-sparing’ approaches [9], and are therefore beyond physiotherapist influence.

Given the age and gender of the population, many patients having radical prostatectomy have, or are at risk of, other, lifestyle-related diseases, e.g. diabetes mellitus, heart disease [10]. As a component of the physiotherapy intervention, patients having radical prostatectomy in our setting are therefore also routinely prescribed general physical activity/exercise. It has been our observation that more physically active patients tend to have reduced, or earlier resolution of, PPUI. Previous studies have shown that increased physical activity is continence protective in other, non-prostate cancer populations [11,12], and a systematic review of preoperative exercise interventions in patients with cancer, including prostate cancer, found both continence and physical capacity benefits [13].

A review of the literature found only one study investigating the effects of preoperative physical activity levels on PPUI [2]. The authors reported that physically active, non-obese patients had a trend towards reduced prevalence of incontinence at 1 year postoperatively compared to inactive, obese counterparts, but there was no significant difference at 6 weeks postoperatively. No physical activity intervention was prescribed to patients in that study. We are interested in the potential for prescribed general physical activity/exercise to reduce PPUI. Therefore in the current, observational study we sought to answer the following questions:

In a cohort of patients receiving a physiotherapist-guided PFM training program and physical activity intervention:

i) How do physical activity levels change over the perioperative period?

ii) What is the relationship between perioperative physical activity levels and PPUI?

Further, we note that robotic-assisted laparoscopic prostatectomy (RALP) purports to facilitate earlier return to normal function/physical activity than open retropubic prostatectomy (ORP) [14]. As both surgical approaches are used in our clinical setting, we also sought to investigate: iii) the effect of surgical approach/group on postoperative physical activity levels and PPUI.

## Methods

This prospective, observational study was undertaken within a urological cancer centre in Western Sydney, Australia. Ethical approval was obtained from Western Sydney Local Health District Human Research Ethics Committee, and all participants provided written informed consent.

### Participants

Between December 2011 and May 2012, patients having radical prostatectomy by one high volume urological cancer surgeon (MIP) and attending physiotherapy for preoperative PFM training were invited to participate. Exclusion criteria included musculoskeletal, neurological and cardiovascular dysfunction precluding unaided mobility.

### Methods

**Figure 1:**
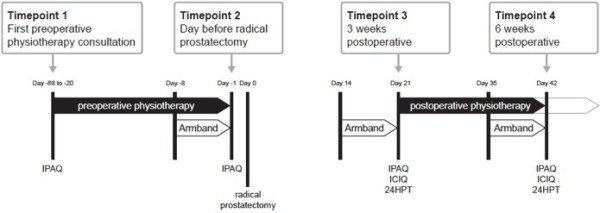
**Study timeline outlining the timing of physiotherapy and assessment procedures in relation to radical prostatectomy (Day 0).***IPAQ*, International Physical Activity Questionnaire; *ICIQ*, International Consultation on Incontinence Questionnaire – Urinary Incontinence Short Form; *24HPT*, 24-hour pad test.

The study timeline is presented in Figure 1. Study participants were assessed at four time-points: at the first preoperative physiotherapy consultation (baseline), on the day before radical prostatectomy, and at 3 and 6 weeks postoperatively, coinciding with routine postoperative physiotherapy consultations.

### Assessment of physical activity

Participants completed the International Physical Activity Questionnaire – Long Form (IPAQ) at all four time-points [15]. The IPAQ is used to estimate physical activity levels over the preceding week. Time spent performing activities of ‘moderate’ and ‘vigorous’ intensity within domains of work, transportation, domestic and gardening activities and leisure is used to calculate two measures of physical activity: i) metabolic equivalent of task (MET) minutes (MET.min/week); and ii) physical activity duration (min/week).

Participants also wore an accelerometer-based physical activity monitor, the SenseWear Pro3 Armband (BodyMedia Inc., Pittsburgh, USA), (henceforth Armband) for three separate periods of one week; the week before surgery and the weeks preceding 3 and 6-week postoperative physiotherapy consultations. The Armband records *inter alia* physical activity intensity (METS) from movement, physiological and anthropometric data using proprietary algorithms [16]. Data were analysed using SenseWear professional software (version 6.1), and two measures of physical activity were derived: i) MET.min/week; and ii) physical activity duration (time >3 METs) (min/week).

The IPAQ categorization protocol was applied to week before surgery physical activity data [17]. This protocol divides respondents into high, moderate and low physical activity categories according to the following algorithm:

i) high: vigorous intensity activity on ≥3 days and accumulating ≥1,500 MET.min/week, or ≥7 days of any physical activity accumulating ≥3,000 MET.min/week;

ii) moderate: vigorous intensity activity on ≥3 days for ≥20 min/day, or moderate intensity activity/walking on ≥5 days for ≥30 min/day, or ≥5 days of any combination of physical activity accumulating ≥600 MET.min/week;

iii) low: any combination of physical activity accumulating <600 MET.min/week.

### Assessment of post-prostatectomy urinary incontinence (PPUI)

PPUI was measured objectively at 3 and 6 weeks postoperatively using a 24-hour pad test (24HPT). Participants were provided with a set of six pre-weighed continence pads, to be worn sequentially over a 24-hour period on the days preceding physiotherapy consultations. Severity of PPUI was calculated as the total weight gain of pads worn.

PPUI was also measured subjectively at 3 and 6 weeks postoperatively using the International Consultation on Incontinence Questionnaire – Urinary Incontinence Short Form (ICIQ) [18]. Questions pertaining to: (i) frequency of urine leakage, (ii) amount of leakage, and (iii) overall impact of leakage were used to calculate a summary score between 0 and 21, greater values indicating increased severity of PPUI.

### Anthropometric and perioperative data

Height and weight were measured using a combined scale/stadiometer (TIWB3000P, Wedderburn, Sydney). Perioperative data, e.g. surgical approach/group, duration of postoperative catheterization, were collected from the surgical record.

### Perioperative physiotherapy

All participants received a standard program of perioperative physiotherapy, consisting of weekly preoperative and 3 and 6-week postoperative appointments. Physiotherapy was provided by one of three men’s health physiotherapists, and included both PFM training and physical activity components. The PFM training program, commenced preoperatively, included: i) patient education regarding the structure and function of the PFMs; ii) supervised contractions of the PFMs in functional positions, e.g. supine lying, sitting and standing; and iii) instruction regarding daily independent practice of contractions at home, including during common activities of daily living. Supervised contractions of the PFMs were undertaken with transabdominal real-time ultrasound feedback; physiotherapists also provided verbal and tactile cues to ensure contractions were performed correctly [6]. Participants also performed stationary cycling and/or treadmill walking exercise of between 15 to 30 min duration during physiotherapy appointments, and were encouraged to walk a minimum of 10,000 steps/day preoperatively.

### Surgical management

All participants had radical prostatectomy in one of four hospitals in Western Sydney. Participants were not randomized to surgical group; the decision for ORP/RALP was predicated on patient preference (including consideration of cost) and the presence of excluding factors, e.g. extensive prior intra-abdominal surgery. ORP was performed as described by Eastham et al [19]. RALP was performed as described by Coelho et al [20], with modified posterior reconstruction of the rhabdosphincter.

### Data analysis

The statistical software package IBM SPSS Statistics Version 20 was used to analyse data. Independent samples t-test and Fisher’s exact test were used to compare surgical groups for anthropometric and perioperative data. Repeated-measures analysis of variance was used to examine change in physical activity over time, and the impact of surgical group and preoperative physical activity category on physical activity levels and PPUI. The Pearson correlation coefficient was used to assess the presence and strength of associations between physical activity levels, physical activity duration and PPUI. Two-tailed tests with a 5% significance level were used throughout. Unless otherwise stated, data are presented as mean (SD).

## Results

**Figure 2:**
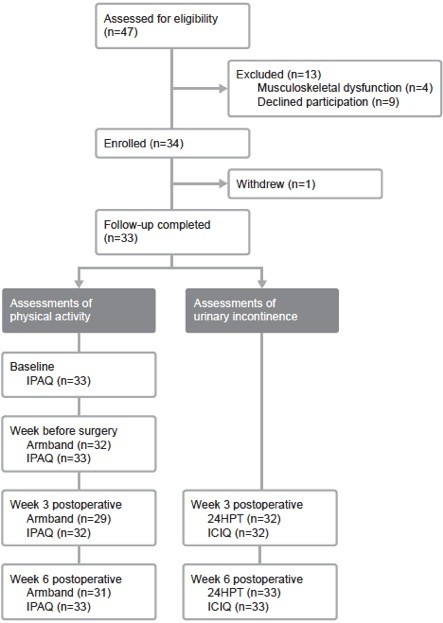
**Flowchart of patients through the study period.***IPAQ*, International Physical Activity Questionnaire; *ICIQ*, International Consultation on Incontinence Questionnaire – Urinary Incontinence Short Form; *24HPT*, 24-hour pad test.

Figure 2 is a flowchart of patients presenting for radical prostatectomy over the study period. Thirty-four patients meeting inclusion criteria consented to participate in the study, one of whom withdrew postoperatively following rehospitalization. His data have been excluded from analysis. Armband data was incomplete for five participants (total six data points) for the following reasons: Armband failure (n = 3); insufficient duration of wear (n = 2); unplanned change in date of surgery (n = 1). One participant did not complete the IPAQ at 3 weeks postoperatively. 24HPT data at 3 weeks postoperatively for one participant was excluded from analysis, as a severe urinary tract infection had resulted in complete loss of urinary control. Participants presented for the first preoperative physiotherapy appointment 39 (11) days preoperatively (range 20–68 days), and attended 5 (1) preoperative physiotherapy appointments (range 3–8). Anthropometric and perioperative data are presented in Table 1.

**Table 1:** Anthropometric and perioperative data for the 33 study participants (mean(SD))

	**ORP (n = 9)**	**RALP (n = 24)**	**Total (n = 33)**
Anthropometric data			
Age (yr)	60 (7)	63 (6)	62 (6)
Height (m)	1.76 (0.07)	1.75 (0.05)	1.75 (0.06)
Weight (kg)	92 (12)	83 (10)	86 (11)
BMI (kg.m^-2^)	29.6 (4.5)	27.3 (3.0)	27.9 (3.6)
Preoperative factors			
PSA (ng/mL)	5.8 (4.2)	5.0 (2.1)	5.2 (2.8)
Preoperative Gleason score			
3 + 3	1 (11%)	5 (21%)	6 (18%)
3 + 4	5 (56%)	16 (67%)	21 (64%)
4 + 3	3 (33%)	2 (9%)	5 (15%)
4 + 4	0 (0%)	1 (4%)	1 (3%)
Clinical tumour stage			
cT1	4 (44%)	13 (54%)	17 (52%)
cT2	5 (56%)	11 (46%)	16 (48%)
cT3	0 (0%)	0 (0%)	0 (0%)
cT4	0 (0%)	0 (0%)	0 (0%)
Prostate volume (cc)	40.2 (13.4)	41.2 (12.5)	40.9 (12.6)
Intraoperative factors			
Nerve sparing			
None	3 (33%)	3 (13%)	6 (18%)
One bundle	2 (22%)	2 (9%)	4 (12%)
Two bundles	4 (44%)	19 (79%)	23 (70%)
Pelvic lymph node dissection	7 (78%)	2 (9%)^a^	9 (27%)
Bladder neck preservation	0 (0%)	23 (96%)^a^	23 (70%)
Postoperative factors			
Postoperative Gleason score			
3 + 3	1 (11%)	3 (13%)	4 (12%)
3 + 4	6 (67%)	16 (67%)	22 (67%)
4 + 3	2 (22%)	5 (21%)	7 (21%)
4 + 4	0 (0%)	0 (0%)	0 (0%)
Pathological tumour stage			
pT2	6 (67%)	18 (75%)	24 (73%)
pT3	3 (33%)	6 (25%)	9 (27%)
pT4	0 (0%)	0 (0%)	0 (0%)
Positive lymph nodes	1/7 (14%)	0/2 (0%)	1/9 (11%)
Positive margins	2 (22%)	2 (9%)	4 (12%)
Duration of postoperative hospital stay (d)	2.9 (0.3)	2.0 (0.2)^a^	2.3 (0.5)
Duration of postoperative catheterization (d)	10.2 (3.0)	8.4 (1.6)	8.9 (2.2)
Anastomic structure	0 (0%)	1 (4%)	1 (3%)

*ORP*, Open retropubic prostatectomy; *RALP*, Robotic-assisted laparoscopic prostatectomy; *BMI*, Body mass index; *PSA*, Prostate specific antigen.

^a^: p < 0.001 vs ORP.

**Figure 3:**
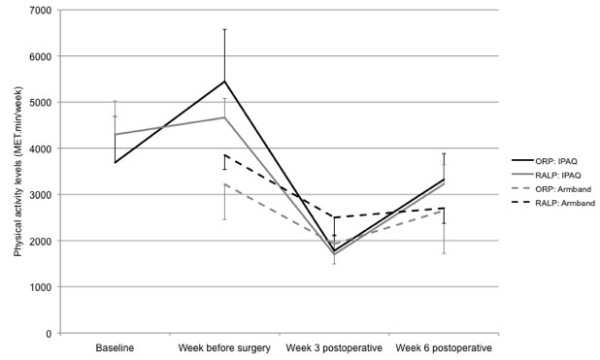
Physical activity levels (MET.min/week) for the 33 study participants (9 ORP, 24 RALP) over the perioperative period, as measured by IPAQ and Armband, separated by surgical group (mean ± SE).

**Figure 4:**
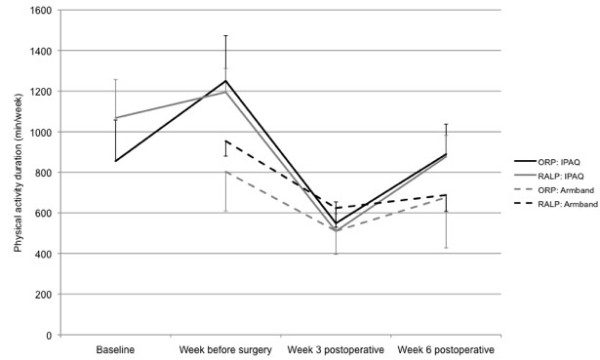
Physical activity duration (min/week) for the 33 study participants (9 ORP, 24 RALP) over the perioperative period, as measured by IPAQ and Armband, separated by surgical group (mean ± SE).

Figures 3 and 4 show physical activity levels and duration over the perioperative period, as measured by both IPAQ and Armband, separated by surgical group. There were no significant differences between surgical groups for baseline or week before surgery physical activity levels (IPAQ: baseline: p = 0.653, week before surgery: p = 0.529; Armband: week before surgery: p = 0.370). There were no significant interaction effects between time and surgical group on physical activity levels (IPAQ: p = 0.832; Armband: p = 0.466).

There was a significant cubic effect of time on IPAQ-measured (subjective) physical activity levels (p < 0.001), i.e. there were two inflexion points in the function of physical activity levels over time. Post-hoc tests showed that subjective physical activity levels did not increase significantly with the preoperative physiotherapy intervention, i.e. from the first, baseline physiotherapy appointment to the week before surgery (p = 0.185), but did decrease significantly below baseline and week before surgery levels at 3 weeks postoperatively (p < 0.001). At 6 weeks postoperatively, subjective physical activity levels did not differ significantly from baseline (p = 0.181), but remained significantly lower than the week before surgery (p = 0.002).

There was a significant quadratic effect of time on Armband-measured (objective) physical activity levels (p = 0.002), i.e. there was one inflexion point in the function of physical activity levels over time. As with subjective physical activity, there was a significant decrease in objective physical activity levels from the week before surgery to 3 weeks postoperatively (p < 0.001). At 6 weeks postoperatively, objective physical activity levels remained significantly lower than the week before surgery (p = 0.002).

Using Armband data from the week before surgery, 18 participants (56%) (3 ORP, 15 RALP) were categorized as having high physical activity levels, and 14 participants (44%) (5 ORP, 9 RALP) were categorized as having moderate physical activity levels (data for one participant (ORP) unavailable). No participants were categorized as having low physical activity levels.

**Figure 5:**
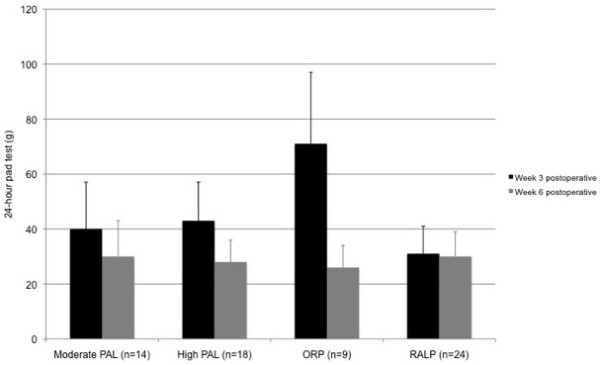
24-hour pad test outcomes at 3 and 6 weeks postoperatively, separated by preoperative physical activity category (moderate vs high physical activity level (PAL), using Armband data from week before surgery) and surgical group (mean + SE).

**Figure 6:**
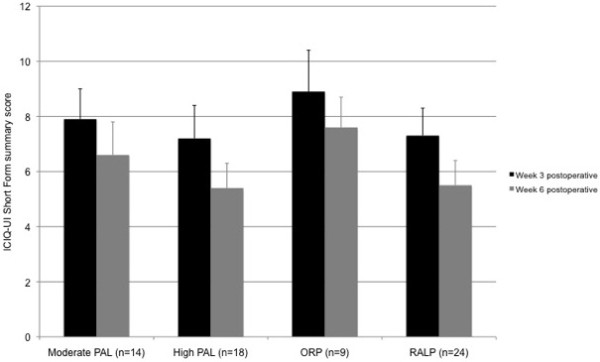
ICIQ-UI Short Form summary score at 3 and 6 weeks postoperatively, separated by preoperative physical activity category (moderate vs high physical activity level (PAL), using Armband data from week before surgery) and surgical group (mean + SE).

Figures 5 and 6 show PPUI outcomes (24HPT and ICIQ) at 3 and 6 weeks postoperatively, separated by preoperative physical activity category and surgical group. There was a significant decrease in PPUI for all participants from 3 to 6 weeks postoperatively (ICIQ: p = 0.001; 24HPT: p = 0.032). There was no significant interaction effect between time and preoperative physical activity category on PPUI (24HPT: p = 0.726; ICIQ: p = 0.608), i.e. preoperative physical activity category did not affect the postoperative time-course of PPUI. Nor were there any significant correlations between absolute preoperative physical activity level (Armband data, week before surgery) and PPUI at 3 weeks (24HPT: r = 0.046, p = 0.805; ICIQ: r = 0.006, p = 0.974) or 6 weeks postoperatively (24HPT: r = −0.073, p = 0.691; ICIQ: r = −0.129, p = 0.483).

Postoperatively, there were no significant correlations between absolute physical activity levels (Armband data) and PPUI at either of 3 weeks (24HPT: r = −0.054, p = 0.779; ICIQ: r = −0.173, p = 0.370) or 6 weeks postoperatively (24HPT: r = 0.070, p = 0.708; ICIQ: r = −0.005, p = 0.978). Similarly, there were no significant correlations between physical activity duration (Armband data) and PPUI at either of 3 weeks (24HPT: r = −0.009, p = 0.965; ICIQ: r = −0.156, p = 0.419) or 6 weeks postoperatively (24HPT: r = 0.054, p = 0.773; ICIQ: r = 0.000, p = 1.000).

There was a significant interaction effect between time and surgical group for the 24HPT (p = 0.044), but not for the ICIQ (p = 0.639), i.e. 24HPT reduced more steeply from week 3 to week 6 postoperatively for participants having ORP. Post-hoc tests showed a non-significant trend (p = 0.090) towards a reduced 24HPT at week 3 postoperatively for participants having RALP, but no significant between group difference at week 6 postoperatively (p = 0.827).

## Discussion

To our knowledge, this is the first study to investigate perioperative physical activity levels in men having radical prostatectomy, and to investigate the relationships between both objectively measured physical activity levels and PPUI. We found that patients receiving a physiotherapist-guided PFMT and physical activity intervention had significantly reduced physical activity levels at 3 weeks postoperatively, but physical activity levels had recovered to baseline (preintervention) levels at 6 weeks postoperatively. There was no significant effect of preoperative physical activity levels on PPUI, nor were there significant correlations between postoperative physical activity levels and severity of PPUI. Finally, surgical group (ORP vs RALP) did not significantly affect the severity of early PPUI, or the course of recovery of postoperative physical activity.

There are limited published data against which to compare our physical activity data. Baseline physical activity as measured with the IPAQ (median 3276 MET.min/week) was similar to that reported in a 12-country (including Australia) study of the IPAQ in healthy adults (median 3699 MET.min/week) [15]. It is suggested that subjective questionnaires, such as the IPAQ, overestimate physical activity levels [21]; as such, from the week before surgery we also used a validated physical activity monitor to objectively measure physical activity levels. Objectively measured physical activity durations from the week before surgery to six weeks postoperatively ranged from 42-66% of those reported in a study of healthy European men (also using the SenseWear Pro3 Armband) [16].

The overall physical activity levels of the cohort must be taken into account when considering the non-relationship between preoperative physical activity levels and PPUI in the current study. The one previously published study reporting an association between inactivity/obesity and PPUI described activity dichotomously (active vs non-active) using a low threshold (</> 1 hour of exercise/week) [2]. By comparison, mean physical activity duration for our cohort in the week before surgery was 15 (7) hours, and no patient did <4 hours of physical activity per week. It is conceivable that a threshold level of preoperative physical activity is continence-protective, and that, by providing our cohort with a physical activity intervention, we ‘lifted’ all patients above that threshold.

The absence of a relationship between postoperative physical activity levels and PPUI is of interest. An *a priori* hypothesis that patients with more severe PPUI might curtail their postoperative physical activity was not supported. Nor did we find evidence that patients engaging in more postoperative physical activity experienced worse PPUI. The clinical implication is that patients might be encouraged to increase their physical activity postoperatively towards (or above) baseline levels, without fear of worsening/delaying return to continence.

The urinary incontinence outcomes support that our cohort had low overall severity of PPUI; this too might have limited the power of the current study to demonstrate significant relationships between perioperative physical activity levels and PPUI. The mode of surgical and perioperative management in the current study (i.e. using a predominantly nerve-sparing approach, all patients receiving preoperative PFMT) has been shown to optimise postprostatectomy continence outcomes [3-5,9]. 24HPTs of 42 ± 59 g at 3 weeks and 29 ± 40 g at 6 weeks postoperatively are at the low end of those reported at similar time-points in trials of PFMT among patients having ORP (median 28–249 g) [22,23], and less than that reported in a cohort of patients receiving RALP with rhabdosphincter reconstruction (184 g at 6 weeks) [24]. Similarly, ICIQs of 8 (5) at 3 weeks and 6 (4) at 6 weeks postoperatively are considerably lower than those reported even in a ‘successful’ randomized trial of preoperative PFMT (15 at 4 weeks postoperatively) [4].

That surgical group did not affect the course of postoperative physical activity was surprising. A key proposed benefit of RALP is that, given the smaller incisions required, patients experience less pain, and ‘faster recovery and return to normal activities’ [14]. Indeed, randomized trials of ORP vs RALP have demonstrated that patients having RALP take less sick leave [25], and have a faster return to baseline quality of life [26]. There are, however inherent difficulties with blinding patients and health practitioners in such trials, and the possibility that that recovery might have been influenced by preconceived patient/practitioner expectations in those studies cannot be discounted. Our results suggest that early return to baseline physical activity levels is feasible regardless of surgical group/approach, given perioperative physiotherapy with a focus on physical activity. Specific surgeon-proscribed activities, e.g. heavy lifting, may still be contraindicated.

### Limitations and strengths of the study

Patients were not randomized to physical activity prescription, therefore the explicit effect of physical activity prescription on PPUI and postoperative physical activity levels cannot be determined. Nor were patients randomized to surgical approach. Physiotherapy including physical activity prescription has been a routine component of the perioperative care pathway for men in our clinical setting, and ethical concerns precluded its withdrawal.

A second limitation of the study is the small sample size, which reduced the power of the study to find significant between-group differences in PPUI and postoperative physical activity levels. Broadening study inclusion criteria to enable recruitment of more participants, and with a greater range of physical activity levels and urinary incontinence outcomes, is perhaps warranted in future studies. The relatively short follow-up period for the study (6 weeks) is not seen as major limitation, as it is uncommon for patients to attend physiotherapy treatment beyond this in our clinical setting, and the benefits of physiotherapy/PFMT for PPUI reduce in the longer term [6].

A strength of the study is the use of both subjective and objective measures of physical activity levels and PPUI. Substantial discrepancies have been shown between subjective and objective measures of PPUI in patients after radical prostatectomy (patients under-reporting PPUI); [27] as with subjective overestimation of physical activity levels these discrepancies may relate to a social desirability bias. Unfortunately, we were unable to obtain a baseline objective measure of physical activity, as preoperative physical activity prescription commenced on the day that patients were recruited to the study (the initial preoperative physiotherapy appointment).

## Conclusions

This study provides novel data on perioperative changes in physical activity levels for men having radical prostatectomy. We found no relationships between perioperative physical activity levels and PPUI, however our cohort had high overall preoperative physical activity levels and low overall PPUI/burden. Surgical group did not affect the course of perioperative physical activity. Randomized trials investigating the effect of preoperative physical activity/exercise on PPUI in exercise-naïve clinical settings are warranted.

## Abbreviations

24HPT: 24-hour pad test; ICIQ: International Consultation on Incontinence Questionnaire – Urinary Incontinence Short Form; IPAQ: International Physical Activity Questionnaire – Long Form; ORP: Open retropubic prostatectomy; PAL: Physical activity level; PFM: Pelvic floor muscle; PPUI: Post-prostatectomy urinary incontinence; RALP: Robotic-assisted laparoscopic prostatectomy; SD: Standard deviation; SE: Standard error.

## Competing interests

The authors declare that they have no competing interests.

## Authors’ contributions

All authors participated in the study conception, design and coordination. SFM, BPH and MIP collected study data. ADH and BPH performed the data analysis. ADH wrote the first draft of the paper with input of all authors. All authors read and approved the final manuscript.

## Pre-publication history

The pre-publication history for this paper can be accessed here:

http://www.biomedcentral.com/1471-2490/13/67/prepub
